# Acute disseminated encephalomyelitis (ADEM) associated with mosquito-borne diseases: Chikungunya virus X yellow fever immunization

**DOI:** 10.1590/0037-8682-0160-2019

**Published:** 2020-01-27

**Authors:** Karolyna Carvalho, Ana Luiza Biancardi, Giovanna Provenzano, Haroldo Moraes

**Affiliations:** 1 Universidade Federal do Rio de Janeiro, Faculdade de Medicina, Departamento de Oftalmologia, Rio de Janeiro, RJ, Brasil.

**Keywords:** Yellow fever, Chikungunya, Acute disseminated encephalomyelitis

## Abstract

Acute disseminated encephalomyelitis (ADEM) is a demyelinating autoimmune neuropathic condition characterized by extensive bilateral and confluent lesions in the cerebral white matter and cerebellum. The basal ganglia and gray matter may also be involved. In most cases, the symptoms are preceded by viral infection or vaccination. In this report, we present a case of ADEM associated with optic neuritis presenting alongside two potential triggering factors: chikungunya virus infection and yellow fever immunization.

## INTRODUCTION

Acute disseminated encephalomyelitis (ADEM) syndrome is a rare demyelinating condition of the central nervous system (CNS), usually diagnosed in children and young adults^1^. Generally, ADEM is an acute monophasic condition associated with magnetic resonance imaging (MRI) findings and a recent history of viral infection or immunization.

Although the pathophysiology of this disease is not completely elucidated, it possibly involves a disruption of the blood-brain barrier, followed by an autoimmune response against myelin proteins and neuronal cells^2^. For the most part, autoimmune manifestations are usually triggered by a preceding infection that leads to an overstimulation of the immune system and autoantibody production in predisposed individuals^3^. Therefore, facing a possible ADEM diagnosis, it is important to confirm the presence of an infectious agent in the CNS. 

The infectious diseases related to ADEM are common childhood viral infections;, such as measles, mumps, and varicella. Other agents have also been reported, including *Epstein-Barr* virus (EBV), *herpes simplex* virus (HSV), *human herpes* virus 6 (HHV-6), *coxsackie* virus, cytomegalovirus (CMV), smallpox, *influenza* A and B, rubella, hepatitis A and B, and *Mycoplasma pneumoniae* (the most common bacterial infection associated with ADEM)^2^ and *chikungunya* virus (CHIKV)

The CHIKV infection is an acute febrile illness associated with arthralgia/arthritis. In 2017, about 185,737 CHIKV symptomatic infections were reported in Brazil^4^. Although, usually a self-limited disease, approximately 40-80% of the cases progress to a chronic phase of musculoskeletal disease^5^. Moreover, atypical presentations occur in up to 1%, including ocular and CNS diseases^6^, such as Guillain-Barré syndrome, myelitis, meningoencephalitis, and various types of encephalomyelitis, such as ADEM syndrome^1^.

Ocular manifestations, although rare, are generally self-limited. Patients major complaints are photophobia, retrobulbar pain, and conjunctivitis-*like* symptoms. Regarding the ophthalmological localization, the anterior segment is one of the main locations, followed by the posterior segment, which may lead to choroiditis, retinitis, and optic neuritis. The involvement of the posterior segment may initiate weeks or even months after the onset of the febrile illness. A retrospective observational analysis of 37 cases of laboratory-confirmed CHIKV revealed that anterior uveitis and optic neuritis, were the main presentations in such infections^8^.

Post-immunization ADEM is commonly associated with certain vaccines composed by inactive or live attenuated virus, such as *influenza* and yellow fever (YF), respectively^9^. The side effects of the YF vaccine include viscerotropic and neurological damage, with myelin as a major target^10^. In Brazil, the incidence of side effects is 0.2 cases per 100,000 vaccine doses, with symptoms typically arising 7 to 21 days post-immunization^10^.

The diagnosis of ADEM is made by clinical exclusion. Most cases, multiple bilateral, asymmetric, and confluent lesions are predominantly observed in the white matter of the CNS. Classically, a peculiarity of ADEM is a radiological steadiness through out its clinical course. Uncommon emergence of new neurological lesions can be associated with relapse episodes. However, it must be highlighted that the appearance of new CNS lesions is a highly suggestive manifestation of multiple sclerosis (MS), which is one of the major differential diagnoses from ADEM syndrome. However, supported by the idea that both alterations (ADEM and MS) share similar physiological mechanism, some authors believe that they fall into the same disease spectrum. Additionally, literature describes that up to 35% of patients primarily diagnosed with ADEM developing criteria for MS over a period of 38 months^2^.

The prognosis of ADEM is commonly benign, having its evolution influenced by the age of the patient, the level of CNS involvement, and the time gap between the symptoms onset and the initiation of the treatment^1^. Patients who present the highest risk of neurological sequelae are elderly individuals and those that maintain the symptoms after treatment. Although there are no established guidelines, treatment consists in the administering of immunosuppressants, mainly intravenous methylprednisolone or dexamethasone. In cases of relapse or unsatisfactory response, use of immunoglobulin and plasmapheresis are indicated^7^.

## CASE REPORT

**FIGURE 1:**
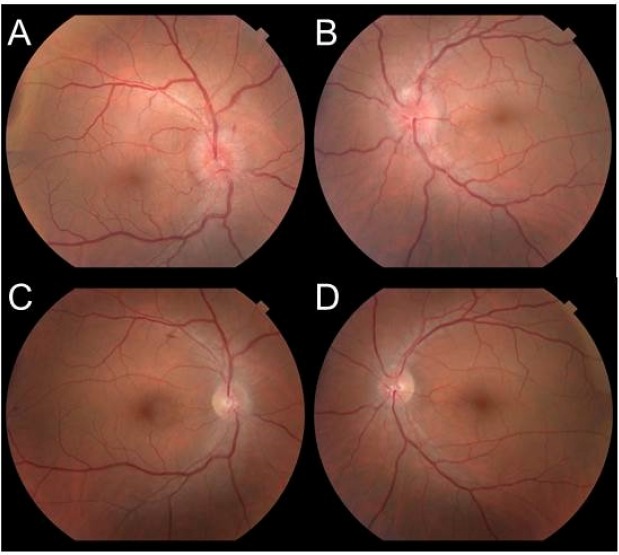
Bilateral color fundus retinography from baseline **(A and B)** to 20 days after treatment **(C and D)**. A and B with optic disc edema in both eyes. C and D with a noted regression of bilateral optic disc edema.

Here, we report the case of a 35-year-old male, unemployed and resident of *São Gonçalo, Rio de Janeiro*. The patient sought ophthalmological care in the uveitis sector of the *University Hospital Clementino Fraga Filho (HUCFF)* in June 14, 2017. The patient reported a visual impairment in both eyes initiated 10 days earlier with no other symptoms or systemic manifestations. No previous comorbidities or medical treatments were reported, with the exception of a recent YF immunization 10 days prior to the onset of visual symptoms. An ophthalmological evaluation revealed the best corrected visual acuity (BCVA) of light perception in both eyes (OU). Biomicroscopy of the anterior segment revealed decreased photomotor reflexes OU, anterior chambers without reaction, anterior vitreous cells OU, and an intraocular pressure of 12/12 mmHg. The fundoscopic exam demonstrated optic disc edema OU (Figure 1A and Figure 1B). Neurological examination revealed a subtle left motor deficit, midline and appendicular ataxia on the left side, and multidirectional nystagmus OU.

The patient was hospitalized and on the basis of the major diagnostic hypothesis, following tests were requested: VDRL, FTA-ABS, C-reactive protein, VHS, anti-HIV and purified protein derivative, anti-aquaporin 4, chest x-ray, and brain and orbit MRI with contrast. The MRI scan revealed multiple subcortical lesions, hyperintense white matter in T2 and FLAIR (fluid-attenuated inversion recovery), hypointensity in T1, increased hypointensity in the center of T2, discrete peripheral enhancement without perilesional edema, and lesions in the cerebellum and midbrain (Figure 1).

In June 16, 2017 a lumbar puncture was performed to collect cerebrospinal fluid (CSF). An examination of the CSF revealed 30 blood cells with 1 leucocyte, 22 mg/dL lactate, 52 mg/dL protein, and 101 mg/dL glucose with no reactive VDRL.

Serological assays were performed to test for the presence of viral agents potentially associated with neuro-ophthalmological alterations, including the YF virus, HSV-1, HSV-2, HHV-6, HHV-7, CMV, EBV, dengue virus (DENV), or zika virus (ZIKV). Among the results, a positive reactivity was exhibit for IgM (immunoglobulin M) against CHIKV. 

Based on the serological outcome, the medical team reassessed the patient and recalled for retrospective data. In May 11, 2017, the subject referred a 1-day, self-limited, unmeasured fever episode associated with headache, myalgia (intense but with partial improvement using common analgesics), and diffuse maculopapular rash with no pruritus. Other symptoms, such as arthralgia, arthritis or edema, conjunctival hyperemia, paranesthesia, and paresis, were not observed. The patient also mentioned a previous clinically suspected arbovirus infections that occurred in his neighborhood in the concomitant period. 

**FIGURE 2:**
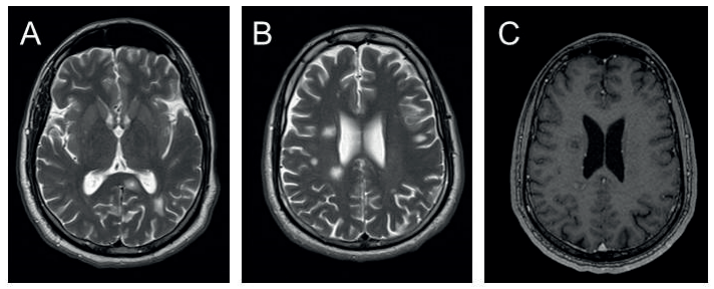
Mass Resonance Imaging - **A and B:** T2 showing numerous oval and rounded lesions disseminated in the subcortical white matter cerebral bilaterally and left cerebellar peduncle. **C:** T1 after contrast with ring enhancement suggesting acute involvement.

Accordingly to the information compiled, it was initiated two cycles of pulsetherapy using 1 mg/kg/day methylprednisolone for 5 and 3 days, respectively. After the initial cycle, the patient’s visual acuity improved to 20/20 OU. Biomicroscopy of the anterior segment was maintained and a complete regression of the optic disc edema OU was observed (Figure 1C and Figure 1D). During this period, the neurological manifestations demonstrated a progressive improvement, concomitantly with the regression of the contrast-enhancing lesions revealed by the MRI report (Figure 2).

In June 23, 2017, the patient presented with left subtle motor deficit, left midline and appendicular ataxia, and multidirectional nystagmus. Subsequently, in June 30, 2017, he presented with ataxic-sensitive gait, cutaneous plantar reflex in left extension, and multidirectional nystagmus. During both episodes, the neurological department chose an observational follow-up, which demonstrated a full neurological recovery. In July 5, 2017, new serum, urine, CSF and anterior chamber humor aqueous samples were collected and tested for antibody or viral RNA from DENV, CHIKV and ZIKV. The serology exam revealed positive for IgM and IgG antibodies for CHIKV and DENV. In comparison, the CSF and urine sample tested by real-time RT-PCR and viral DNA, respectively, were negative, along with the aqueous humor. 

## DISCUSSION

In the above case, the findings were compatible with those described for ADEM. The patient presented with an abrupt onset of visual impairment, discrete motor and balance alterations, a multidirectional nystagmus, characteristic MRI findings and two potential provoking previous infections. 

The laboratory results suggested an immuno-mediated pathological diagnosis, possibly triggered by hyperstimulation after viral infection. Cerny *et al*. showed that neurological presentations occur, on average, 10 days after the onset of the classic symptoms of infection. Up to 20% of these presentations remained asymptomatic between the onset of symptoms and the onset of neurological complaints^2^.

Corroborating this evaluation, the patient presented with an asymptomatic interval between non-specific infection and ophthalmological manifestations. The infection was self-limited and preceded a highly complex ophthalmological and neurological presentation, characterized by a low viremia 30 days after the onset of the condition. Another relevant and confusing factor was the YF vaccine (FA-17DD) preceding the ADEM onset. It is important to highlight that the acquired immune response verified by attenuated vaccines starts approximately 07 days post-immunization, demonstrating a antibody peak over the 14th day. In this case report, a temporal relationship could be associated between the vaccine shot and the neuro- ophthalmological manifestations. However, no antibody response for YF was detected, possibly dismissing this diagnosis.
